# LINC00266-1/miR-548c-3p/SMAD2 feedback loop stimulates the development of osteosarcoma

**DOI:** 10.1038/s41419-020-02764-8

**Published:** 2020-07-24

**Authors:** Shengnai Zheng, Li Wan, Dawei Ge, Fan Jiang, Zhanyang Qian, Jian Tang, Jin Yang, Yilun Yao, Junwei Yan, Lei Zhao, Haijun Li, Lei Yang

**Affiliations:** 1 Department of Orthopedic Surgery, Nanjing First Hospital, Nanjing Medical University, 210006 Nanjing, Jiangsu China; 2 Department of Oncology, The Affiliated Huai’an No.1 People’s Hospital of Nanjing Medical University, 223300 Huai’an, Jiangsu China; 3 Department of Orthopedics, The First Affiliated Hospital of Nanjing Medical University, 210029 Nanjing, Jiangsu China; 4 Burn and Plastic Surgery, The First Affiliated Hospital of Nanjing Medical University, 210029 Nanjing, Jiangsu China; 5 Department of Pathology, Wuxi Third People’s Hospital, 214000 Wuxi City, Jiangsu China; 6 Department of Orthopedics, Taizhou People’s Hospital Affiliated to Nantong University, 225300 Taizhou, Jiangsu China

**Keywords:** RNA, Bone cancer

## Abstract

Osteosarcoma (OS) is one of the most common primary bone malignancies and accounts for 3.4% of pediatric tumors. Its 5-year survival is as low as about 20%. Differentially expressed lncRNAs in OS profiling were searched in the downloaded profile of GSE12865. As a result, LINC00266-1 was detected to be upregulated in both GSE12865 and OS tissues we collected. SMAD2 was the downstream target binding to promoter sites of LINC00266-1, displaying a positive regulatory interaction. Knockdown of LINC00266-1 suppressed the proliferative and metastatic abilities, and promoted the apoptosis in OS cells. Besides, knockdown of LINC00266-1 significantly alleviated the growth of OS in vivo. MiR-548c-3p was the sponge miRNA of LINC00266-1, which was able to reverse the regulatory effects of LINC00266-1 on OS cell phenotypes. Moreover, miR-548c-3p bound to the 3′-UTR of SMAD2 and thus downregulated SMAD2. Overexpression of SMAD2 partially reversed the regulatory effects of LINC00266-1 on OS cell phenotypes. Finally, we have identified that LINC00266-1/miR-548c-3p/SMAD2 feedback loop was responsible for stimulating the development of OS.

## Introduction

Osteosarcoma (OS) is a prevalent primary bone malignancy that mainly affects adolescents. Osteosarcoma accounts for 3.4% of pediatric tumor cases^1^. Although surgery combined with chemotherapy has been widely applied for the treatment of OS, the 5-year survival rate of OS is only 20% because of its potent invasiveness, high metastatic rate and recurrence^2^. It is necessary to explore the key mechanisms underlying the development of OS, thus improving the therapeutic efficacy of its treatments and its prognosis.

With the development of whole-genome sequencing technology, it was determined that protein-coding sequences account for less than 2% of the human genome^3^. LncRNAs (long noncoding RNAs) are noncoding RNAs that contain over 200 nucleotides^4^. Although they rarely encode proteins, they are extensively involved in gene expression regulation, chromosome remodeling, transcription modulation, posttranscriptional processing, etc.^5,6^. Differentially expressed lncRNAs in human cancers have been identified^7,8^. To date, only a small number of lncRNAs have been discovered^9^. In recent years, diagnostic or prognostic potential of lncRNAs in OS has been well concerned. For example, MEG3 is closely linked to overall survival in OS, serving as a prognostic marker^10^. Knockdown of MFI2 can suppress the proliferative and metastatic abilities in OS cells^11^. However, the biological functions of lncRNAs in OS tumorigenesis have not been well characterized.

A newly proposed theory suggested that a single lncRNA can inactivate biological functions of multiple miRNAs by sponging them^12^. MiRNAs are small, noncoding RNAs containing 17–25 nucleotides that post-transcriptionally regulate gene expressions through complementary base pairing, thus inhibiting the translation of target mRNAs and protein accumulation^13,14^. They are involved in tumorigenesis and tumor development^15^. Many dysregulated miRNAs responsible for triggering the invasiveness of OS cells have been discovered^16,17^. MiRNA-based targeted therapies may be effective strategies for OS.

LINC00266-1, also known as NCRNA00266-1, is located on chromosome 20 (GRCh38.p13). Its specific function in OS, however, has rarely been reported. In our present study, we found that SMAD2 can activate the expression of LINC00266-1 by binding to its promoter sequence. In addition, LINC00266-1 was upregulated in OS tissues compared with the corresponding nontumor tissues. It regulated proliferative, migratory and apoptotic potentials in OS cells. The important role of LINC00266-1 in the tumorigenesis of OS was dependent on miR-548c-3p/SMAD2 axis.

## Results

### LINC00266-1 was upregulated in OS

**Fig. 1:**
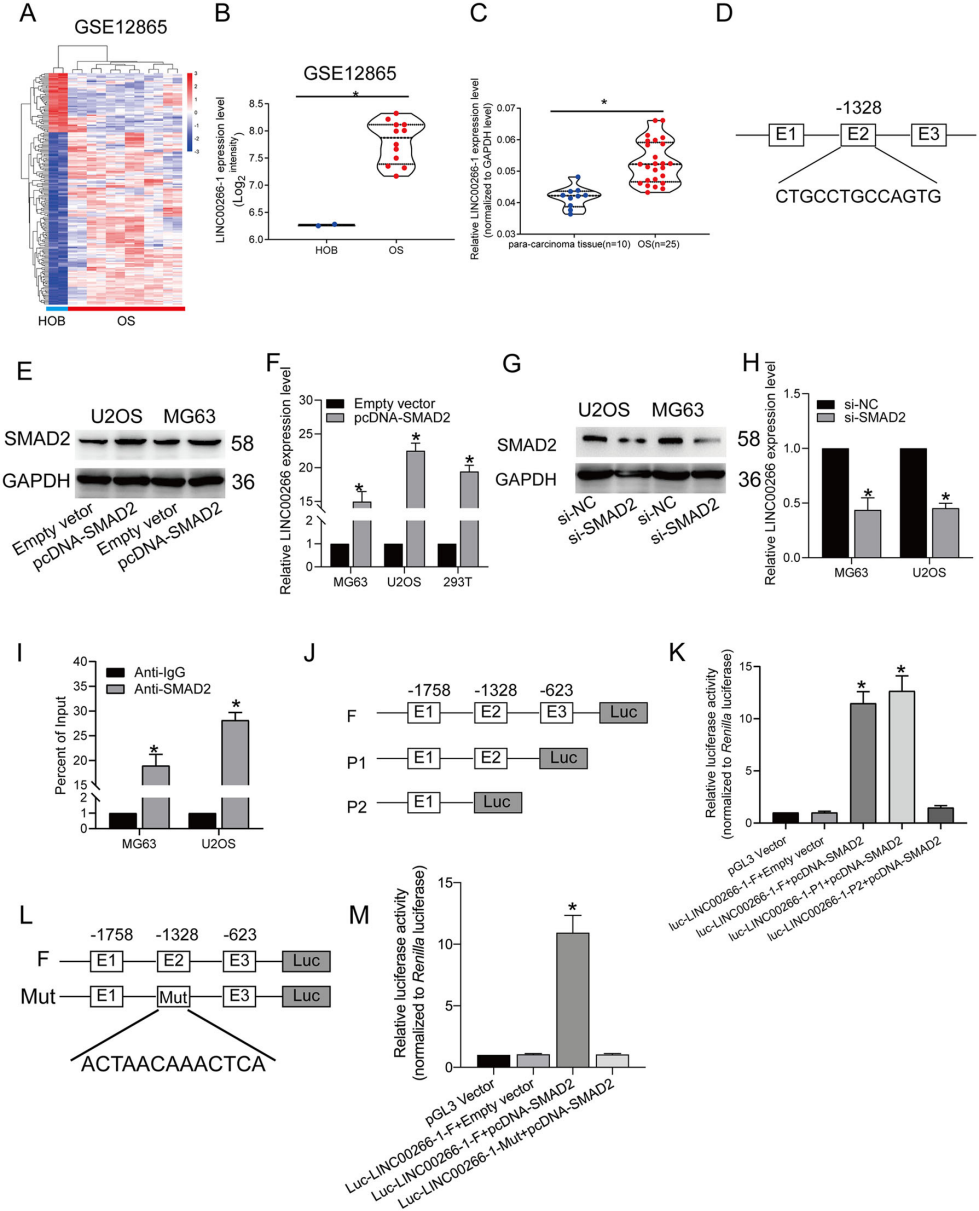
SMAD2 bound the promoter region of LINC00266-1. **a**, **b** LINC00266-1 was upregulated in OS tissues analyzed in the GSE12865 dataset. **c** LINC00266-1 was upregulated in OS tissues. **d** Three binding sites for SMAD2 in the upstream region of LINC00266-1 were predicted by Jaspar. **e** Overexpression of SMAD2 upregulated the protein expression of SMAD2 in MG63 and U2OS cells. **f** Overexpression of SMAD2 upregulated LINC00266-1 in MG63, U2OS and 293T cells. **g** Knockdown of SMAD2 downregulated the protein expression of SMAD2 in MG63 and U2OS cells. **h** Knockdown of SMAD2 downregulated LINC00266-1 in MG63, U2OS and 293T cells. **i** ChIP assay revealed the binding of SMAD2 to the promoter region of LINC00266-1. **j**, **k** Dual-luciferase reporter assay revealed that SMAD2 bound in the region 1328 nucleotides upstream of LINC00266-1. **l**, **m** The luciferase reporter plasmid with the mutation sequence of E2 markedly reduced the luciferase activity in the Mutation sequence. *p < 0.05.

In order to obtain the dysregulated lncRNAs, the OS gene expression data were downloaded (GSE12865). The signal data were normalized, and *z*-score-transformed. We mainly focused on upregulated lncRNAs because they may be more readily used as early diagnostic markers or therapeutic targets. In our research, LINC00266-1 was found to be upregulated in OS in GSE12865 (Fig. 1a, b). To confirm this finding, we examined LINC00266-1 levels in OS tissues collected in our hospital. Consistent with the in silico analysis results, LINC00266-1 was highly expressed in OS tissues (*n* = 10) compared with adjacent nontumor tissues (*n* = 25) (Fig. 1c).

### Transcription factor SMAD2 activated LINC00266-1 transcription in OS cells

Transcription of lncRNAs is regulated by transcription factors such as p53, SP1, and E2F1. To investigate the potential transcriptional regulators involved in LINC00266-1 overexpression in OS, we first scanned for potential transcription factors that share binding sites in the LINC00266-1 promoter. SMAD2 was identified using online bioinformatics tool (http://jaspar.genereg.net) and there were three potential binding sites in the promotor sites (Fig. 1d). Thus, we hypothesized that SMAD2 is involved in the transcription that leads to lncRNA LINC00266-1 overexpression. In order to confirm the hypothesis overexpression of SMAD2 by transfection with pcDNA-SMAD2 into MG63 and U2OS cells could effectively upregulate the protein level of SMAD2, which confirmed the transfection efficacy firstly (Fig. 1e). Moreover, overexpression of SMAD2 upregulated LINC00266-1 in MG63, U2OS and 293T cells as well (Fig. 1f). Similarly, the transfection efficiency of si-SMAD2 was verified (Fig. 1g). Knockdown of SMAD2 downregulated LINC00266-1 in OS cells (Fig. 1h). Then, the binding of SMAD2 to the promoter region of LINC00266-1 was confirmed by ChIP assay (Fig. 1i). As shown in Fig. 1j, three promoter regions, designated as Luc-LINC00266-1-F (−400 to −2000), Luc-LINC00266-1-P1 (−100 to −2000), and Luc-LINC00266-1-P2 (−1500 to −2000), were cloned into a luciferase reporter plasmid to ensure the SMAD2-binding sites (Fig. 1j). MG63 cells were cotransfected with the pcDNA-SMAD2/Empty-vector and Luc-LINC00266-1-F, Luc-LINC00266-1-P1 and Luc-LINC00266-1-P2. The luciferase activity was markedly increased in the former two groups, rather than Luc-LINC00266-1-P2. This result revealed that SMAD2 bound the E2 region located on the 1328 nucleotides upstream of LINC00266-1 (Fig. 1j, k). Furthermore, we also constructed a luciferase reporter plasmid, containing the mutation sequence of E2. Then we cotransfected the pcDNA-SMAD2/Empty-vector and Luc-LINC00266-1-F, Luc-LINC00266-Mut. The result showed that the luciferase activity markedly reduced in the mutation sequence (Fig. 1l, m).

### Knockdown of LINC00266-1 suppressed proliferative and metastatic abilities, and induced apoptosis in OS cells

**Fig. 2:**
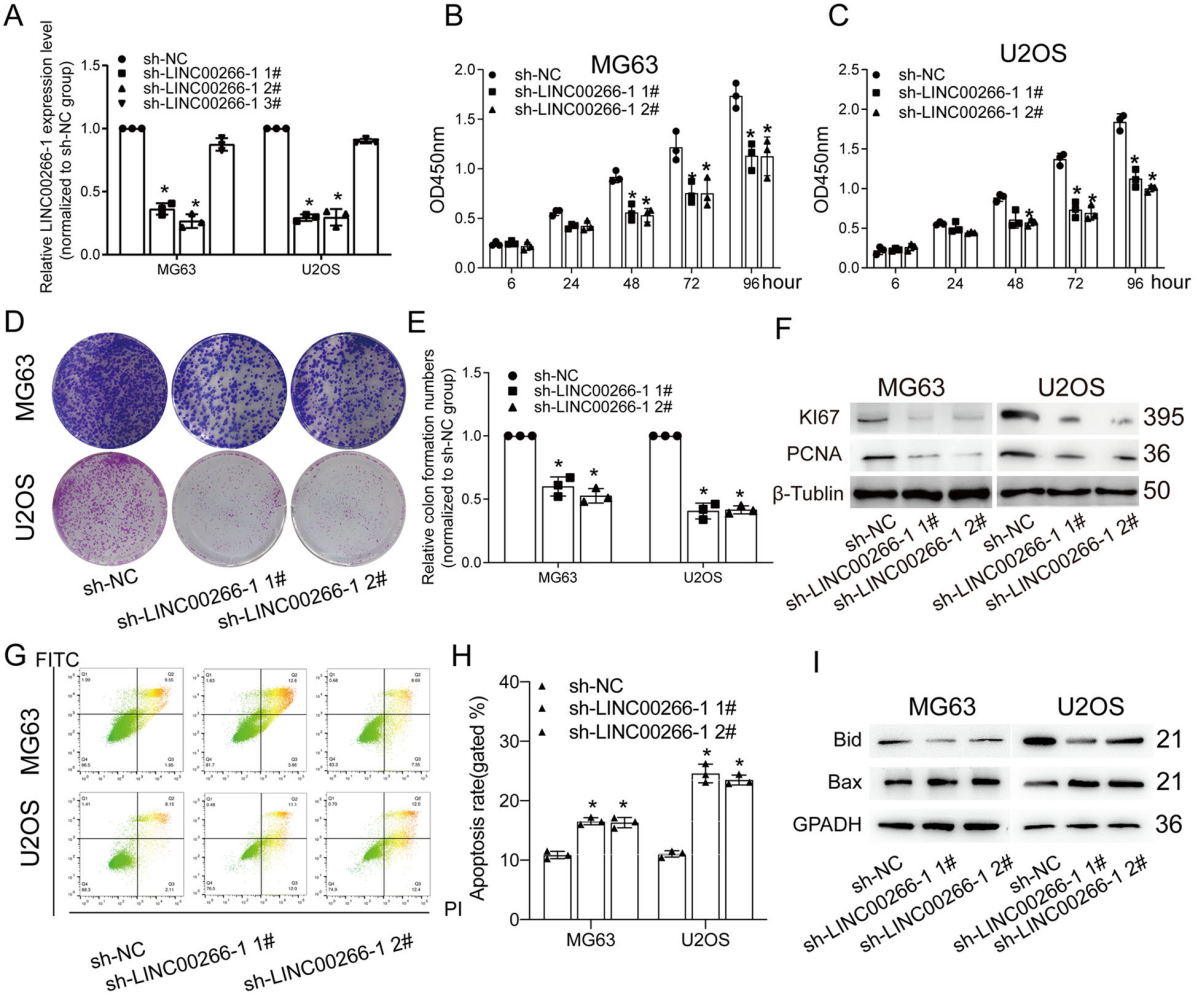
Knockdown of LINC00266-1 suppressed the proliferative ability and induced the apoptosis in OS cells. **a** Transfection of sh-LINC00266-1 #1 or sh-LINC00266-1 #2 downregulated LINC00266-1 in MG63 and U2OS cells. **b**, **c** CCK-8 assay showed that knockdown of LINC00266-1 reduced the viability in MG63 and U2OS cells. **d**, **e** Colony formation assay showed that knockdown of LINC00266-1 reduced the colony formation ability in MG63 and U2OS cells. **f** Protein levels of Ki67 and PCNA were downregulated in MG63 and U2OS cells with LINC00266-1 knockdown. **g**, **h** Flow cytometry showed that knockdown of LINC00266-1 increased the apoptotic rate in MG63 and U2OS cells. **i** Knockdown of LINC00266-1 downregulated Bid and upregulated Bax in MG63 and U2OS cells. *p < 0.05.

**Fig. 3:**
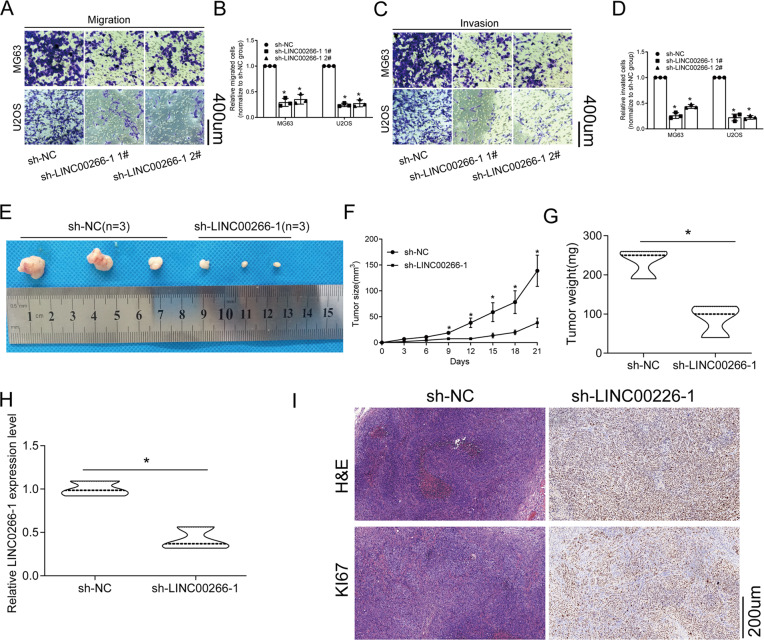
Knockdown of LINC00266-1 reduced the migratory and invasive abilities in OS cells and inhibited the growth of OS in vivo. **a**, **b** Transwell assay showed that knockdown of LINC00266-1 suppressed the migration in MG63 and U2OS cells. **c**, **d** Transwell assay showed that knockdown of LINC00266-1 suppressed the invasion in MG63 and U2OS cells. **e** OS tissues were collected from nude mice 21 days after cell administration. **f** Mice administered with MG63 cells transfected with sh-LINC00266-1 #1 presented slower tumor growth than control mice. **g** The tumor weight was lower in mice administered with MG63 cells transfected with sh-LINC00266-1 #1 than in control mice. **h** The in vivo level of LINC00266-1 decreased in OS tissues harvested from mice administered with MG63 cells transfected with sh-LINC00266-1 #1. **i** IHC showed lower positive expression of Ki67 in mice administered with MG63 cells transfected with sh-LINC00266-1 #1 than in control mice. *p < 0.05.

The biological functions of LINC00266-1 in regulating cancer cell phenotypes remain unclear. Further exploration of the biological function of LINC00266-1 in the progression of OS involved three short hairpin RNA (sh-LINC00266-11#, sh-LINC00266-12#, sh-LINC00266-12#) against LINC00266-1 and the nontargeting shRNA (sh-NC) were designed and stable LINC00266-1 knockdown MG63 and U2OS cell lines were established. The knockdown efficiency was evaluated by qRT-PCR (Fig. 2a). The cell counting kit-8 (CCK-8) assay (Fig. 2b, c), colony formation assay (Fig. 2d, e) and EdU assay (Supplementary Fig. 1A, B) showed that knockdown of KCNQ1OT1 partly inhibited the proliferation of MG63 and U2OS cells. We next examined the expression levels of proliferation-associated proteins in OS cells. Protein expressions of Ki67 and PCNA were downregulated in OS cells with LINC00266-1 knockdown (Fig. 2f). Furthermore, the potential influence of LINC00266-1 on cell apoptosis was assessed by flow cytometry and TUNEL staining analysis. As depicted in Fig. 2g–i, flow cytometry (Fig. 2g–i) and TUNEL assay (Supplementary Fig. 1C, D) demonstrated that the apoptosis rate was much higher after silencing LINC00266-1 than the sh-NC group. Besides, downregulated Bid and upregulated Bax after knockdown of LINC00266-1 supported our findings that LINC00266-1 inhibited apoptosis in OS cells (Fig. 2m). The migratory (Fig. 3a, b) and invasive (Fig. 3c, d) abilities in OS cells were weakened by knockdown of LINC00266-1. Collectively, the downregulation of LINC00266-1 silenced proliferative and metastatic abilities, whereas induced apoptosis in OS cells, serving as an oncogenic role.

### Knockdown of LINC00266-1 inhibited the growth of OS in vivo

Subsequently, nude mice bearing OS xenografts were prepared for analyzing the in vivo influence of LINC00266-1 on the growth of OS. Nude mice were subcutaneously administrated with the MG63 cells which were transfected with sh-NC or sh-LINC00266-1 1# for 48 h. Twenty-one days later, mice administered with MG63 cells transfected with sh-LINC00266-1 1# presented slower tumor growth than those of controls (Fig. 3e, f). Mice were sacrificed for collecting OS tissues. The tumor weight was much lower in the sh-LINC00266-11# group than the sh-NC group (Fig. 3g). In addition, we found that LINC00266-1 expression remained relatively lower in OS tissues harvested from mice sh-LINC00266-1 1# (Fig. 3h). IHC showed decreased positive expression of Ki67 after knockdown of LINC00266-1 in nude mice (Fig. 3i). These findings indicated that knockdown of LINC00266-1 inhibited the growth of OS in vivo.

### LncRNA LINC00266-1 functioned as a ceRNA to sponge miR-548c-3p

**Fig. 4:**
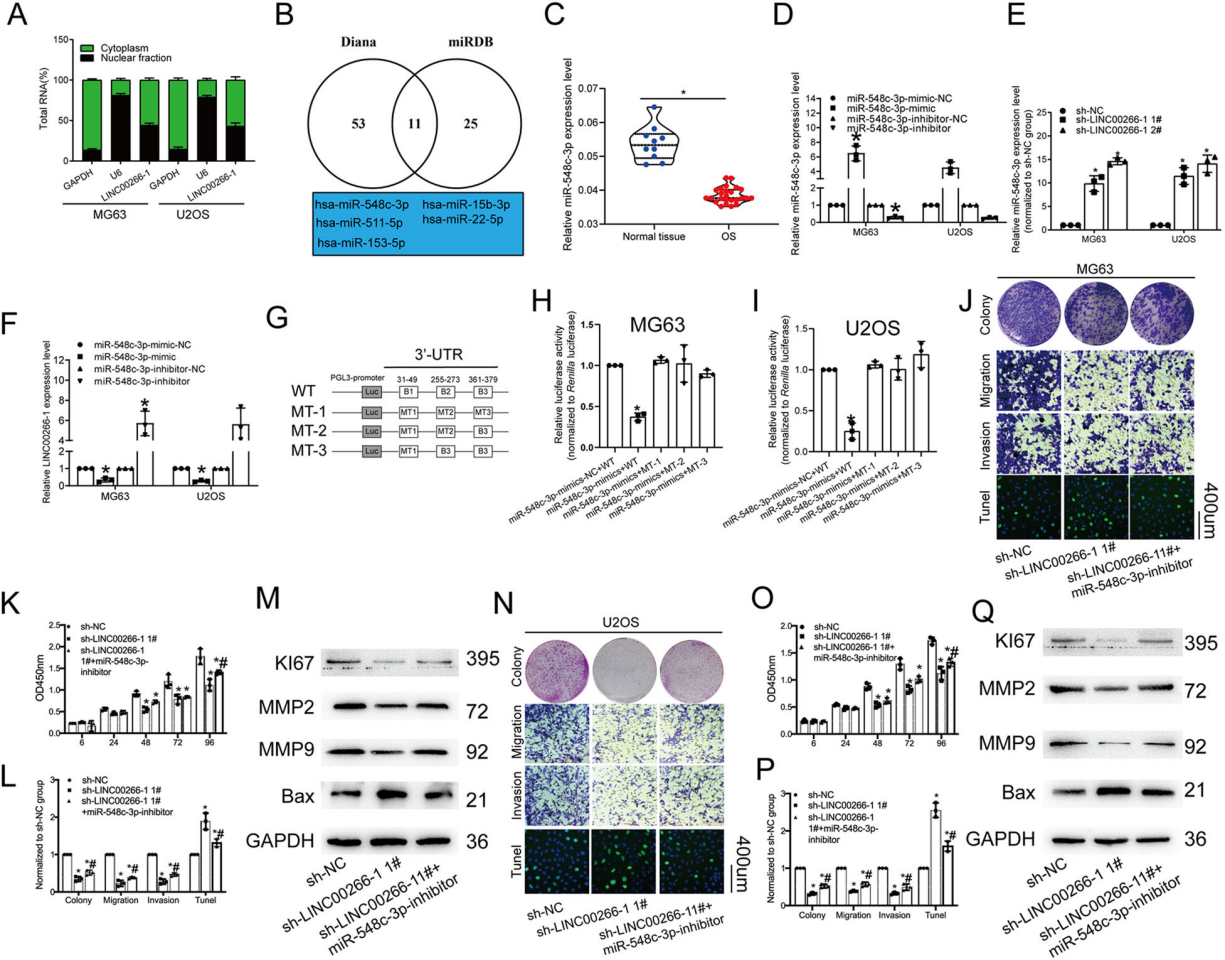
LINC00266-1 sponged miR-548c-3p and miR-548c-3p abolished the effect of LINC00266-1 on OS cell phenotypes. **a** LINC00266-1 was distributed in both the cytoplasm and nuclei. **b** Eleven potential miRNAs binding LINC00266-1 were searched in the Diana and miRDB databases. Literature review showed that five miRNAs have been extensively explored. **c** MiR-548c-3p was downregulated in OS tissues. **d** Transfection efficiency of miR-548c-3p mimic and inhibitor. **e** MiR-548c-3p was upregulated in OS cells with LINC00266-1 knockdown. **f** LINC00266-1 was downregulated by overexpression of miR-548c-3p, and it was upregulated by knockdown of miR-548c-3p. **g**–**i** Dual-luciferase reporter assay showed that the B1 mutation failed to affect the luciferase activity in MT-1, MT-2 and MT-3 in MG63 and U2OS cells. **j**–**l** The proliferation, metastasis and apoptosis in MG63 cells were influenced by miR-548c-3p and LINC00266-1. **m** Relative levels of Bax, Ki67, MMP2 and MMP9 in MG63 cells were influenced by miR-548c-3p and LINC00266-1. **n**–**p** The proliferation, metastasis and apoptosis in U2OS cells were influenced by miR-548c-3p and LINC00266-1. **q** Relative levels of Bax, Ki67, MMP2 and MMP9 in U2OS cells were influenced by miR-548c-3p and LINC00266-1 (*p < 0.05. ^#^*p* < 0.05, compared to sh-LINC00266-1 1# group).

To uncover the potential mechanism of LINC00266-1 in OS development, subcellular distribution analysis was conducted in OS cells. The results showed that LINC00266-1 was distributed in both the cytoplasm and nucleus (Fig. 4a), indicating that LINC00266-1 exerted both transcriptional and posttranscriptional modification. LncRNAs can function as miRNA sponges and thus regulate target gene expressions. To confirm our hypothesis, we predicted 11 potential miRNAs as the targets of LINC00266-1 in the Diana and miRDB databases. In order to narrow the scope, five miRNAs (miR-548c-3p, miR-15b-3p, miR-511-3p, miR-22-5p and miR-153-5p) have been extensively explored on their functions in tumor progression through literature review (Fig. 4b). Among the five candidates, we found that miR-548c-3p was markedly downregulated in OS tissues (Fig. 4c). We constructed miR-548c-3p mimic and inhibitor, and confirmed their knockdown efficacy in MG63 and U2OS cells (Fig. 4d). Besides, miR-548c-3p was upregulated in OS cells with LINC00266-1 knockdown (Fig. 4e). Additionally, LINC00266-1 level was negatively regulated by miR-548c-3p (Fig. 4f). As predicted, there were three binding sites of miR-548-3p in the 3′-UTR region of LINC00266-1. To explore the exact binding site, we conducted the luciferase plasmid containing the whole wild-type sequence (WT) of LINC00266-1. Furthermore, we also constructed three luciferase plasmids harboring three mutated binding sites (MT-1), two mutated sites (MT-2) and one mutated site (MT-3), respectively. Dual-luciferase reporter assay showed that the B1 mutation failed to affect the luciferase activity in MT-1, MT-2 and MT-3 in both MG63 and U2OS cells, suggesting that miR-548c-3p could bind to the B1 region which was located from the 31−49 nuclear of 3′-UTR of LINC00266-1 (Fig. 4g–i). Collectively, LINC00266-1 specifically bound to miR-548c-3p and negatively regulated its level. Interestingly, knockdown of miR-548c-3p abolished the regulatory effects of silenced LINC00266-1 on suppressing the proliferative and metastatic abilities, and stimulating apoptosis in MG63 cells (Fig. 4j–l). We previously showed that knockdown of LINC00266-1 upregulated Bax and downregulated Ki67, MMP2 and MMP9 in MG63 cells. The above trends were reversed by knockdown of miR-548c-3p (Fig. 4m). Similar results were obtained in U2SO cells (Fig. 4n–q). The above results showed that LINC00266-1 regulated OS cell phenotypes by acting as a ceRNA to sponge miR-548c-3p.

### SMAD2 was the target of miR-548c-3p

**Fig. 5:**
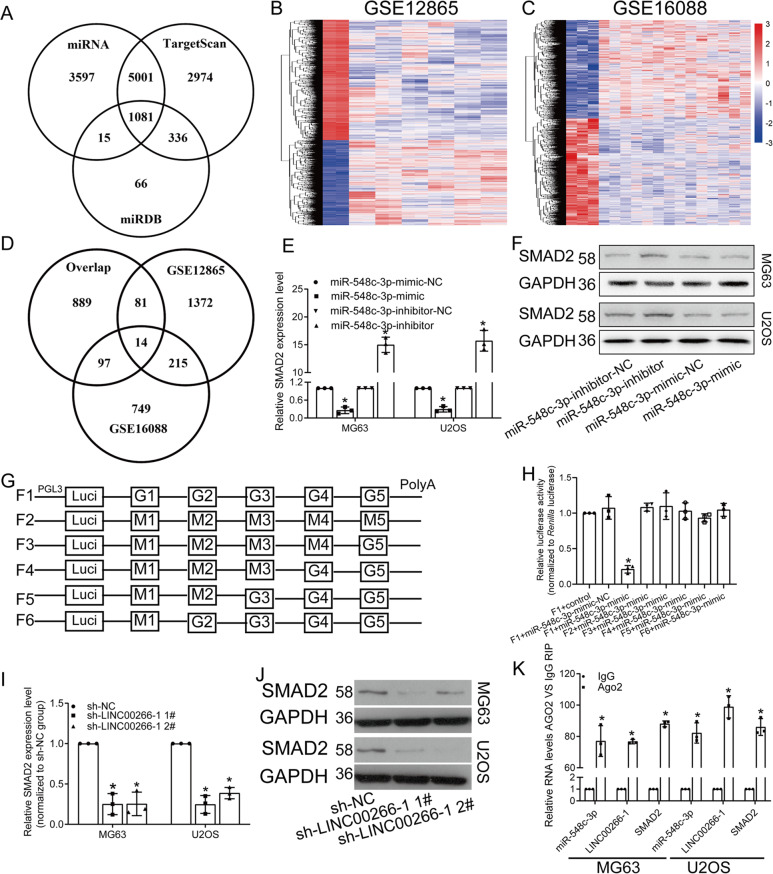
MiR-548c-3p downregulated SMAD2 by binding it. **a** A total of 1081 genes were searched as the downstream targets of miR-548c-3p in miRNA, TargetScan and miRDB databases. **b**, **c** Differentially expressed mRNAs in OS tissues from the GSE12865 and GSE16088 datasets. **d** Fourteen upregulated mRNAs were selected. **e**, **f** SMAD2 was upregulated by knockdown of miR-548c-3p, and it was downregulated by overexpression of miR-548c-3p. **g**, **h** Dual-luciferase reporter assay showed that miR-548c-3p bound the G1 region of SMAD2. **i**, **j** Knockdown of LINC00266-1 decreased the mRNA and protein levels of SMAD2. **k** RIP assay showed that LINC00266-1, miR-548c-3p and SMAD2 were enriched in AGO2. *p < 0.05.

As previously reported, miRNAs bind to the 3′UTR of target genes and then downregulate them. Potential target genes of miR-548c-3p were predicted using miRNA, TargetScan and miRDB. A total of 1081 candidates were searched (Fig. 5a). To narrow down the range of target genes, we downloaded the expression profile of OS including GSE12865 and GSE16088 from GEO datasets. Differentially expressed mRNAs in OS tissues were analyzed in both datasets (Fig. 5b, c). Then, we get the overlap among the dysregulated mRNAs in GSE12865 and GSE16088 and the predicted candidates. As a result, 14 mRNAs were selected, and we ultimately focused on SMAD2 (Fig. 5d). Then, we found that both mRNA and protein levels of SMAD2 were negatively regulated by miR-548c-3p (Fig. 5e, f). As predicted, there were five binding sites located on the 3′-UTR of SMAD2. We thereafter constructed the luciferase plasmid containing whole wild-type sequence (F1). Besides, the sequences containing mutated sites were cloned into luciferase plasmid respectively (F2, F3, F4, F5, F6). As dual-luciferase reporter assay results showed, miR-548c-3p bound to the G1 region of SMAD2 (Fig. 5g, h). Furthermore, knockdown of LINC00266-1 markedly downregulated the mRNA and protein levels of SMAD2 (Fig. 5i, j). To clarify the interactions among LINC00266-1, miR-548c-3p and SMAD2, RIP assay was conducted. The data revealed that all three genes were enriched in AGO2 (Fig. 5k).

### Overexpression of SMAD2 abolished biological functions of LINC00266-1 in regulating OC cell phenotypes

**Fig. 6:**
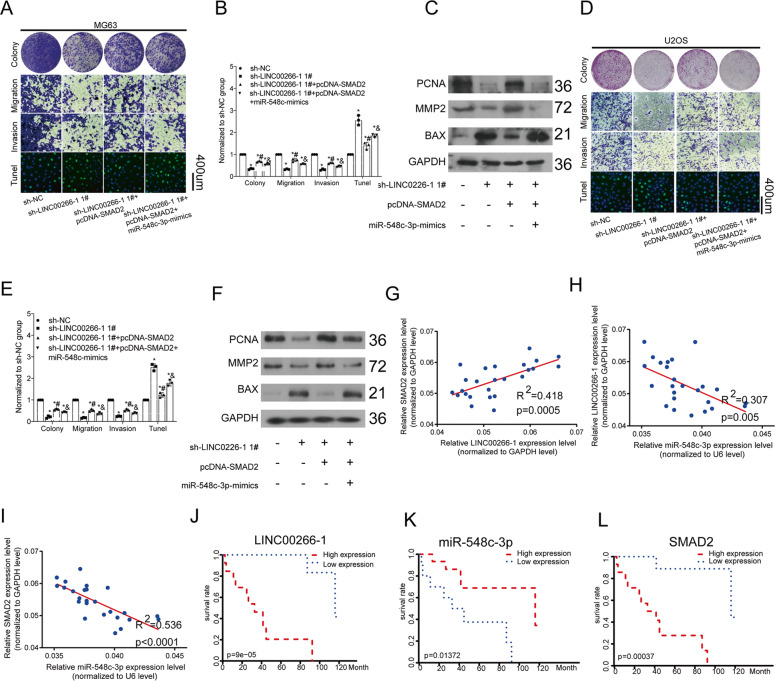
Overexpression of SMAD2 abolished the role of LINC00266-1 in OS development. **a**, **b** The proliferation, metastasis and apoptosis in MG63 cells were influenced by LINC00266-1, miR-548c-3p and SMAD2. **c** Relative levels of Bax, MMP2 and PCNA in MG63 cells were influenced by LINC00266-1, miR-548c-3p and SMAD2. **d**, **e** The proliferation, metastasis and apoptosis in U2OS cells were influenced by LINC00266-1, miR-548c-3p and SMAD2. **f** Relative levels of Bax, MMP2 and PCNA in U2OS cells were influenced by LINC00266-1, miR-548c-3p and SMAD2. **g**–**i** The LINC00266-1 level was positively correlated with that of SMAD2 and negatively correlated with that of miR-548c-3p. The SMAD2 level was negatively correlated with that of miR-548c-3p. **j**–**l** LINC00266-1 and SMAD2 were favorable to the prognosis in OS, and miR-548c-3p was an unfavorable factor (*p < 0.05. ^#^*p* < 0.05, compared to sh-LINC00266-1 1# group, ^&^*p* < 0.05, compared to sh-LINC00266-1 1#+pcDNA-SMAD2 group).

We have already proved that knockdown of LINC00266-1 inhibited proliferative and metastatic abilities, as well as induced apoptosis in MG63 cells. The above effects were reversed by overexpression of SMAD2, and interestingly, enhanced by overexpression of miR-548c-3p (Fig. 6a, b). Cotransfection of sh-LINC00266-1 and pcDNA-SMAD2 downregulated Bax and upregulated both MMP2 and PCNA, which were reversed by overexpression of miR-548c-3p (Fig. 6c). Similar results were obtained in U2OS cells (Fig. 6d–f). Hence, overexpression of SMAD2 partially rescued the effects of LINC00266-1 on OS development.

### Prognostic potentials of LINC00266-1, miR-548c-3p and SMAD2 in OS

Finally, we explore the correlation among the three factors and their relationship with prognosis. Pearson correlation analysis revealed that LINC00266-1 level was positively correlated with that of SMAD2 and negatively correlated with that of miR-548c-3p. In addition, the SMAD2 level was negatively correlated with that of miR-548c-3p (Fig. 6g–i). Survival analysis revealed that LINC00266-1 and SMAD2 were unfavorable to the prognosis in OS, while miR-548c-3p was a favorable factor (Fig. 6j–l).

## Discussion

Although great progress has been made in OS treatment, its overall survival has not been greatly improved^18^. It is urgent to clarify the mechanisms underlying the development, metastasis and recurrence of OS. Critical roles of lncRNAs in tumor development have been highlighted^19,20^. Multiple lncRNAs, such as HOXD-AS1, HOTAIR, SNHG5 and MALAT-1, are abnormally expressed in OS samples, exerting potential regulatory effects^21–24^. In this paper, we first demonstrated that LINC00266-1 was highly expressed in OS tissues. In vitro and in vivo experiments indicated that knockdown of LINC00266-1 suppressed the proliferative and metastatic abilities and induced the apoptosis in OS cells. Our findings suggested that LINC00266-1 exerted a carcinogenic role during the development of OS. It has been reported that lncRNAs are vital regulators in tumorigenesis through mediating chromosome modification, transcription modulation and miRNA sponging^25,26^. In addition, a subset of lncRNAs are enriched in the cytoplasm, where they participate in cellular biological processes by functioning as ceRNAs or regulating mRNA stability, mRNA alternative splicing and protein localization^27^.

In our research, we found that LINC00226-1 was mainly localized in the cytoplasm. Then, we searched the target miRNAs of LINC00266-1 in Diana and miRDB datasets. Through 11 total targets searched, five of them have been extensively researched. Previous studies have shown the involvement of miR-548c-3p in many types of cancer^28–30^. Luo et al.^31^ demonstrated that miR-548c-3p is downregulated in OS. Knockdown of miR-548c-3p drives tumor development by stimulating tumor cell growth, inhibiting apoptosis and arresting the cell cycle in G2/M phase. Consistently, we found that miR-548c-3p was lowly expressed in OS tissues and its level was negatively regulated by LINC00266-1. Notably, miR-548c-3p abolished the regulatory effects of LINC00266-1 on OS cell phenotypes.

MiRNAs are critical regulators by targeting to the 3′UTR of mRNAs, thus forming a complicated network involved in tumor development^32^. In a similar way, 1081 genes were predicted to be potential targets of miR-548-3p. After screening differentially expressed mRNAs in OS profiling of GSE12865 and GSE16088, SMAD2 was finally selected to be specifically explored. SMAD2 is an important factor in the TGF-β pathway. Binding between TGF-β ligands and receptors phosphorylates SMAD2 and SMAD3. Subsequently, SMAD2/3 and SMAD4 complexes are translocated into the nucleus, where they directly bind SMAD-binding elements and further mediate tumor cell phenotypes^33–35^. Our findings confirmed that SMAD2 was the direct target of miR-548c-3p. Its level was negatively regulated by miR-548c-3p and positively regulated by LINC00266-1. Furthermore, RIP assay demonstrated that LINC00266-1, miR-548c-3p and SMAD2 were enriched in AGO2. Rescue experiments finally confirmed the involvement of the LINC00266-1/miR-548c-3p/SMAD2 feedback loop in stimulating the development of OS.

In summary, we demonstrated for the first time that LINC00266-1 is upregulated in OS, serving a carcinogenic role in promoting the proliferative and metastatic abilities in OS cells through the miR-548c-3p/SMAD2 feedback loop. Our findings provide potential OS biomarkers for developing therapeutic strategies. However, the conclusion obtained in this study should be further validated in in vivo experiments in detail.

## Materials and methods

### Sample collection

Osteosarcoma tissues and nontumor tissues were collected from OS patients treated in our hospital. Tissue samples were immediately frozen in liquid nitrogen and stored at −80 °C.

### Cell culture and transfection

OS cell lines (MG63 and U2OS) and the 293T cell line were purchased from the Institute of Biochemistry and Cell Biology, Shanghai. Dulbecco’s modified eagle medium (Thermo Fisher Scientific, Waltham, MA, USA) containing 10% fetal bovine serum (FBS) (Gibco, Rockville, MD, USA) and 1% penicillin-streptomycin was used for cell culture in a 5% CO_2_ incubator at 37 °C. Cell passage was conducted at 90% confluence. Fresh medium was replaced in an interval of 2−3 days. Transfection plasmids (sh-LINC00266-1 #1, sh-LINC00266-1 #2, sh-LINC00266-1 #3, miR-548c-3p-mimic, miR-548c-3p inhibitor, negative control, si-NC, si-SMAD2, pcDNA-NC and pcDNA-SMAD2) were provided by GenePharma (Shanghai, China). Cells in the logarithmic growth phase were selected for transfection using Lipofectamine 2000 (Invitrogen).

### Cell counting kit (CCK-8) assay

2 × 10^3^ cells per well were inoculated in 96-well plates (Corning, Shanghai, China). Using the CCK-8 kit (Dojindo Laboratories, Kumamoto, Japan), viability curves were depicted based on the recorded 450 nm absorbance (*A*).

### Quantitative real-time polymerase chain reaction (qRT-PCR)

TRIzol (Invitrogen, Carlsbad, CA, USA) was used for extracting RNAs, which were reversely transcribed to cDNA using the PrimeScript RT kit (Takara, Dalian, China) after determination of concentration and purity. qRT-PCR was conducted using the SYBR Green method (Takara). Relative level was calculated using the 2^−ΔCT^ method, and normalized to that of GAPDH or U6. The primer sequences were: GAPDH-F: 5′-ATCACCATCTTCCAGGAGGGA-3′, GAPDH-R: 5′-CCTTCTCCATGG TGGTGAAGAC-3′; U6-F: 5′-CTCGCTTCGGCAGCACA-3′ and U6-R: 5′-AACGCTTCACGAATTTGCGT-3′; MiR-548c-3p-F: 5′-ACACTCCAGCTGGGCAAAAATCTCAAT-3′, MiR-548c-3p-R: 5′-CTCAACTGGTGTCGTGGA-3′; SMAD2-F: 5′-AATACGGTAGATCAGTGGGACA-3′, SMAD2-R: 5′-CAGTTTTCGATTGCCTTGAGC-3′.

### Transwell assay

Cells (3 × 10^4^) cultured in serum-free medium were inoculated in the upper chamber of Transwell inserts (8 μM pore size; Corning Incorporated, NY, USA) precoated with Matrigel. A total of 500 μL of complete medium was applied to the bottom. After 48 h of cell culture, penetrating cells were subjected to crystal violet staining. The number of penetrating cells was counted in five randomly selected fields under a microscope.

### Western blotting

Radioimmunoprecipitation assay (Beyotime, Shanghai, China) was used for lysing cells or tissues. The mixture was centrifuged at 4 °C, 12,000 × *g* for 5 min. Protein samples were quantified using the BCA assay. Ten micrograms protein sample was loaded in 15% SDS-PAGE and transferred to polyvinylidene fluoride membranes. After nonspecific antigen blockage, the membrane was immunoprecipitated with primary antibodies (ProteinTech, Wuhan, China) at 4 °C overnight and secondary antibodies for 2 h. Band exposure was achieved by electrochemiluminescence (Pierce, Rockford, IL, USA) and analyzed by Image-Pro Plus (Media Cybernetics, Silver Springs, MD, USA).

### Colony formation assay

Five hundred cells per well were inoculated in a six-well plate. Culture medium was replaced in an interval of 3 days. Visible colonies following 2-week culture were fixed, dyed with 0.05% crystal violet. Visible colonies were manually counted. The number of colonies was normalized to sh-NC group.

### 5-Ethynyl-2′-deoxyuridine (EdU) assay

50 μmol/L EdU was used to label cells at 37 °C for 2 h. Cells were subjected to 30-min fixation in 4% paraformaldehyde, 20-min incubation with 0.5% Triton X-100 and washed in phosphate buffered saline (PBS) containing 3% bovine serum albumin. Subsequently, cells were dyed in 100 μL of staining solution per well for 1 h in the dark, and counterstained with 1× Hoechst 33342 for 30 min. Images of EdU-positive cells and DAPI-labeled nuclei, as well as merged images, were acquired under a microscope (magnification, ×100).

### Flow cytometry

Cells were double-stained with Annexin V-FITC/PI (Kaiji Biological, Inc., Shanghai, China) and subjected to flow cytometry. The apoptosis rate was analyzed with ModFit LT software.

### TUNEL

Cells were subjected to a 30-min fixation with 4% paraformaldehyde, followed by a 30-min incubation in H_2_O_2_ to inactivate endogenous enzymes. Cells were immersed in 0.2% Triton X-100 solution for 5 min to enhance cell membrane permeability and were further incubated with deoxynucleotide terminal transferase (rTdT) at 37 °C for 1 h. Nuclei were stained brown. Five fields were randomly selected from each section. The apoptosis rate was finally calculated (magnification, ×100).

### Chromatin immunoprecipitation (ChIP)

Cells were crosslinked in 1% methanol, lysed, and fragmented to a size of 250−500 bp. They were incubated with an anti-E2F1 antibody or IgG. Subsequently, the chromosome−antibody complexes were separated on Protein A/G Plus-Agarose. Immunoprecipitates were purified and used for qRT-PCR.

### RNA immunoprecipitation (RIP)

Cell lysates were incubated with the antibody at 4 °C for 6 h. Captured protein−RNA complexes were digested in 0.5 mg/mL proteinase K containing 0.1% SDS. The magnetic beads were repeatedly washed in RIP wash buffer (Millipore, Billerica, MA). Finally, qRT-PCR was used to determine the enrichment of the extracted RNA.

### Dual-luciferase reporter assay

Luciferase vectors were constructed based on the predicted binding sequences and transfected into cells, with pRL-TK as the control for transfection efficiency. Forty-eight hours later, cells were collected for measuring luciferase activity using the dual-luciferase reporter system (Promega, WI, USA).

### Tumorigenesis assay

Tumorigenesis assay was approved by the Experimental Animal Center of Nanjing Medical University. Six male BALB/c nude mice (three per group), 4 weeks old (JSJ-Lab, Shanghai, China), were acclimated in a standard environment (22−26 °C, 40−70% humidity) with food and water and without specific pathogen. For the in vivo proliferation assay, MG63 cells were transfected with small hairpin RNA (sh-LINC00266-1 1#) and sh-NC and harvested from six-well plates, washed with PBS, and resuspended at a density 1 × 10^7^ cells/mL. Each mouse was subsequently injected in the lower right flank with 100 μL of suspended cells. Then the transfected cells were administrated into nude mice. Nude mice were subcutaneously administrated with MG63 cells transfected with sh-NC or sh-LINC00266-1 1 for 48 h. Tumor volume was recorded every 3 days and calculated: Volume (mm^3^) = 1/2 (length × width). Mice were killed to harvest tumors 21 days later.

### Immunohistochemistry (IHC)

Mouse OS tissues were harvested and fixed in 4% paraformaldehyde, embedded in paraffin and sectioned. Sections were blocked and incubated with an anti-Ki67 antibody. Finally, immunohistochemical staining was developed using a diaminobenzidine substrate, and sections were counterstained with hematoxylin.

### Statistical analysis

SPSS20.0 software (SPSS IBM, Armonk, NY USA) and GraphPad 7.0 (La Jolla, CA, USA) were processed for data analyses. Data were expressed as means ± standard deviations. Pearson correlation test was conducted to assess the correlation between the expression of two genes. Differences between groups were analyzed using the Student’s *t* test. Multigroup comparison was done using one-way ANOVA test, followed by post hoc test (Least Significant Difference). Significant difference was set at *p* < 0.05.

## Supplementary information

Supplementary Information

Supplementary Information

## Data Availability

The datasets used and/or analyzed during the current study are available from the corresponding author on reasonable request.
