# Supplementary material for: LINC00266-1/miR-548c-3p/SMAD2 feedback loop stimulates the development of osteosarcoma
Source: Cell Death Dis. 2020 Jul 24;11(7):576. doi: 10.1038/s41419-020-02764-8 (PMC7381647; doi:10.1038/s41419-020-02764-8)
Supplement: Supplementary file 1 — Supplementary Information [file 41419_2020_2764_MOESM1_ESM.docx]

**Supplementary Fig 1. A, B** EdU assay showed that knockdown of LINC00266-1 reduced the ratio of EdU-positive MG63 and U2OS cells. **C, D** TUNEL assay showed that knockdown of LINC00266-1 stimulated the formation of apoptotic bodies in MG63 and U2OS cells.
