# Supplementary figures and images for: LINC00266-1/miR-548c-3p/SMAD2 feedback loop stimulates the development of osteosarcoma
Source: Cell Death Dis. 2020 Jul 24;11(7):576. doi: 10.1038/s41419-020-02764-8 (PMC7381647; doi:10.1038/s41419-020-02764-8)

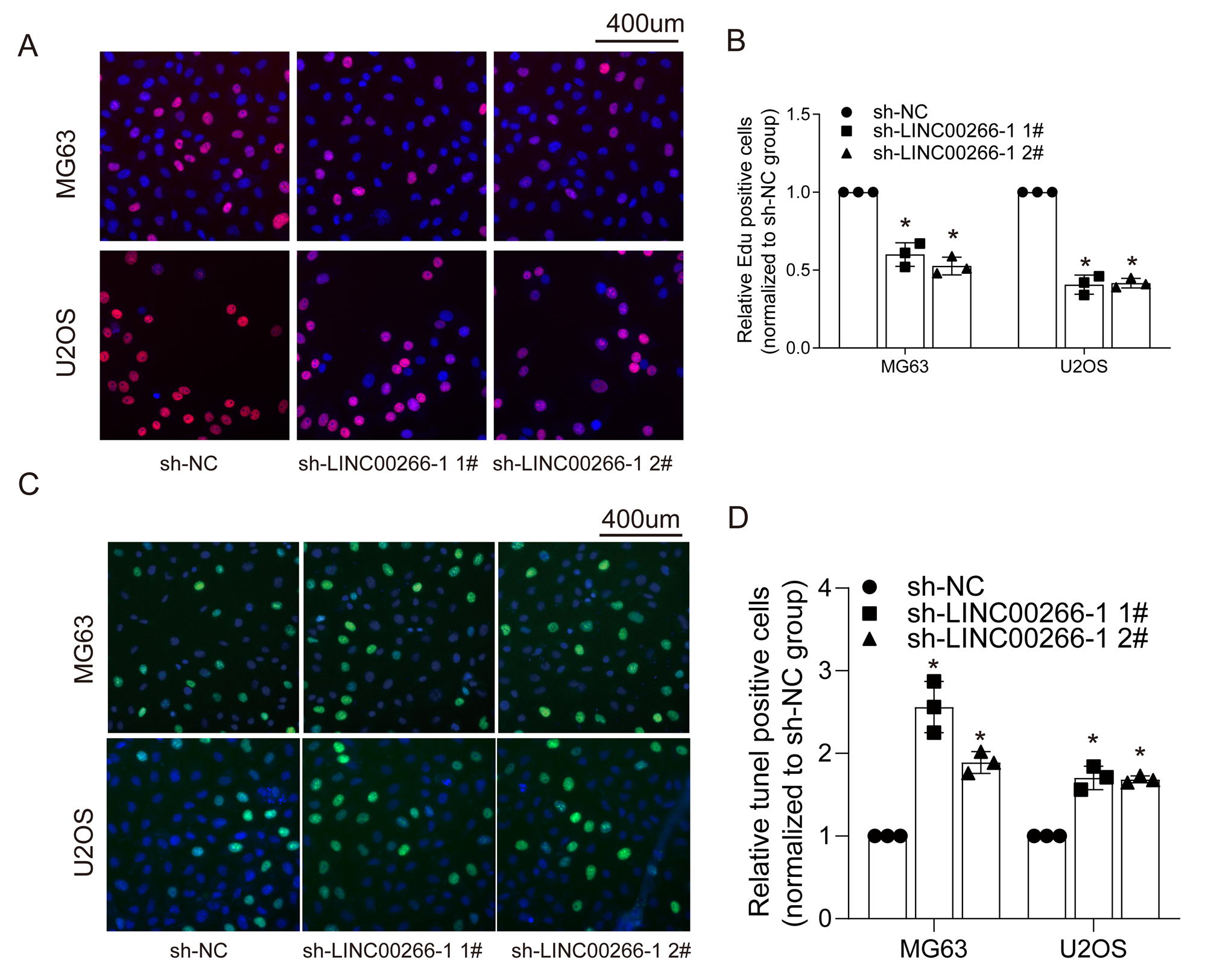

Supplement: Supplementary file 2 — Supplementary Information [file 41419_2020_2764_MOESM2_ESM.tif]
